# An Accessible Clinical Decision Support System to Curtail Anesthetic Greenhouse Gases in a Large Health Network: Implementation Study

**DOI:** 10.2196/40831

**Published:** 2022-12-08

**Authors:** Priya Ramaswamy, Aalap Shah, Rishi Kothari, Nina Schloemerkemper, Emily Methangkool, Amalia Aleck, Anne Shapiro, Rakhi Dayal, Charlotte Young, Jon Spinner, Carly Deibler, Kaiyi Wang, David Robinowitz, Seema Gandhi

**Affiliations:** 1 Department of Anesthesia and Perioperative Care University of California, San Francisco San Francisco, CA United States; 2 Department of Anesthesiology and Perioperative Care University of California, Irvine Irvine, CA United States; 3 Department of Anesthesiology and Pain Medicine University of California, Davis Sacramento, CA United States; 4 Department of Anesthesiology and Perioperative Medicine University of California, Los Angeles Los Angeles, CA United States; 5 Department of Anesthesiology University of California, San Diego San Diego, CA United States; 6 School of Medicine University of California, San Francisco San Francisco, CA United States; 7 San Francisco Medical Center University of California San Francisco, CA United States

**Keywords:** clinical decision support, sustainability, intraoperative, perioperative, anesthetic gas, waste reduction, fresh gas flow

## Abstract

**Background:**

Inhaled anesthetics in the operating room are potent greenhouse gases and are a key contributor to carbon emissions from health care facilities. Real-time clinical decision support (CDS) systems lower anesthetic gas waste by prompting anesthesia professionals to reduce fresh gas flow (FGF) when a set threshold is exceeded. However, previous CDS systems have relied on proprietary or highly customized anesthesia information management systems, significantly reducing other institutions’ accessibility to the technology and thus limiting overall environmental benefit.

**Objective:**

In 2018, a CDS system that lowers anesthetic gas waste using methods that can be easily adopted by other institutions was developed at the University of California San Francisco (UCSF). This study aims to facilitate wider uptake of our CDS system and further reduce gas waste by describing the implementation of the FGF CDS toolkit at UCSF and the subsequent implementation at other medical campuses within the University of California Health network.

**Methods:**

We developed a noninterruptive active CDS system to alert anesthesia professionals when FGF rates exceeded 0.7 L per minute for common volatile anesthetics. The implementation process at UCSF was documented and assembled into an informational toolkit to aid in the integration of the CDS system at other health care institutions. Before implementation, presentation-based education initiatives were used to disseminate information regarding the safety of low FGF use and its relationship to environmental sustainability. Our FGF CDS toolkit consisted of 4 main components for implementation: sustainability-focused education of anesthesia professionals, hardware integration of the CDS technology, software build of the CDS system, and data reporting of measured outcomes.

**Results:**

The FGF CDS system was successfully deployed at 5 University of California Health network campuses. Four of the institutions are independent from the institution that created the CDS system. The CDS system was deployed at each facility using the FGF CDS toolkit, which describes the main components of the technology and implementation. Each campus made modifications to the CDS tool to best suit their institution, emphasizing the versatility and adoptability of the technology and implementation framework.

**Conclusions:**

It has previously been shown that the FGF CDS system reduces anesthetic gas waste, leading to environmental and fiscal benefits. Here, we demonstrate that the CDS system can be transferred to other medical facilities using our toolkit for implementation, making the technology and associated benefits globally accessible to advance mitigation of health care–related emissions.

## Introduction

### Background

In recent years, the medical community has come to widely recognize the health impacts of climate change and its own critical contribution and role in turning back the tide toward a safe and healthy planet [1]. Notably, the health care sector accounts for 8.5% of all greenhouse gas (GHG) emissions in the United States [2]. Volatile anesthetic agents are potent GHGs and contribute up to 5% of the total carbon dioxide (CO_2_) emissions of the National Health Service in the United Kingdom and >50% of the surgical emissions in North America [3,4]. The most commonly used volatile anesthetic agents—sevoflurane, isoflurane, and desflurane—have global warming potentials that are 130 to 2540 times greater than the global warming potential of CO_2_ on a 100-year time horizon [5]. By safely reducing fresh gas flow (FGF), the carrier for anesthetic gases, we can decrease the waste and environmental impact of volatile anesthetic agents while simultaneously reducing cost per case [6-8].

Anesthesia professionals in the United States have historically avoided using low FGF rates because of theoretical safety concerns regarding the accumulation of compound A, a byproduct of sevoflurane processing by CO_2_ absorbents, which has shown nephrotoxic effects in animal models [9]. However, substantial research was never able to replicate these results in human studies, thus invalidating the concern, and the European Common Market never adopted these low-flow guidelines [10]. Furthermore, the new generation of CO_2_ absorbents lacks strong hydroxide bases and thus does not produce compound A. Research has shown that the use of low FGF is safe and effective [11,12]. Considering that the US Food and Drug Administration still recommends maintaining FGF at >2 L per minute with sevoflurane to minimize the production of compound A, low FGF with sevoflurane is currently considered an *off-label* practice [13]. Attitudes on the safety of low FGF rates have evolved, but institution-level behavioral modifications have been difficult to achieve without the proper tools [14]. Educational initiatives alone infrequently result in sustained behavioral change [15], whereas point-of-care visual reminders can promote changes in anesthesia professional behavior that reduce anesthetic gas waste [16]. However, maintenance of these initiatives requires significant time and effort, which are scarce resources in a high-capacity hospital.

### Implementation of an FGF Clinical Decision Support System

Electronic clinical decision support (CDS) systems, which enhance clinical decision-making with real-time prompts and reminders [17], can also help enact behavioral change. The ideal CDS system is accurate, concise, flexible, easy to use, and imparts a minimal cognitive load [18]. Previous studies have demonstrated the utility of CDS systems to optimize anesthetic care and patient safety [19]. Directed CDS alerts have been shown to improve clinician compliance with reducing FGF [20]. Of note, previous CDS tools have lacked generalizability and portability because of reliance on heavily customized proprietary anesthesia information management systems (AIMSs). Deploying CDS systems can be challenging and must be grounded in the mission of an organization, not just the IT systems [21]. To date, no formalized and widely deployable FGF CDS alert has been expanded across different health systems, lessening the global impact of the technology. Recently, a CDS system within a commercial electronic health record (EHR) was developed and validated by the University of California San Francisco (UCSF) Medical Center [22]. A validation study by Olmos et al [22] at UCSF demonstrated that the CDS system effectively reduced FGF rates, volatile anesthetic consumption, and financial costs in the operating room (OR) and that the effects were sustained beyond a year after implementation. In this study, we describe the implementation of the FGF CDS system at UCSF, with subsequent implementation across the University of California (UC) Health network, a system that has pledged to reach carbon neutrality by 2025 [23]. We accomplished this objective by sharing a portable framework, or toolkit, whose core elements are compatible with most commercially available and proprietary AIMSs. Specifically, our implementation study describes the following aspects:

- The detailed technical framework to build, deploy, and track a CDS alert that prompts anesthesia professionals to lower FGF rates (notable FGF CDS system terms and definitions are described in Textbox 1)
- The management guidance to facilitate the integration of the CDS tool into clinical practice through education initiatives
- The launch timeline and characteristics of each FGF CDS system implemented by individual UC Health systems

**Notable terms and definitions for understanding the fresh gas flow clinical decision support toolkit implementation.**

- Global warming potential
  - The amount of energy the emissions of 1 ton of a gas will absorb over a given period in years compared with the emissions of 1 ton of carbon dioxide
- Minimum alveolar concentration (MAC)
  - The minimum concentration of an inhaled anesthetic present in alveoli at 1 standard atmosphere of atmospheric pressure that prevents skeletal muscle movement in response to a surgical incision in 50% of patients; MAC values are used to compare potency among inhaled anesthetic agents
  - The concentration of inhaled anesthetic required to achieve this end point decreases with age [24]
- Fresh gas flow rate
  - The total volume of gas that flows from the anesthetic machine into the breathing system per minute; fresh gas flow serves as the carrier for volatile anesthetic gases
- MAC-hour
  - The average MAC during a treatment period multiplied by the duration of treatment in hours
  - Does not fully encapsulate inhalational anesthetic use, which also depends on fresh gas flow rate
- Best Practice Advisory
  - The brand name for rule-based clinical decision support alerts within the Epic electronic health record (Epic Systems Corporation)
- Middleware
  - A device integration solution for capturing and transmitting anesthesia ventilator data (and other physiological data) to the electronic health record (eg, Capsule Medical Device Integration Platform [Capsule Technologies, Inc, a subsidiary of Philips Healthcare])

## Methods

### Project Approval and Launch

**Figure 1:**
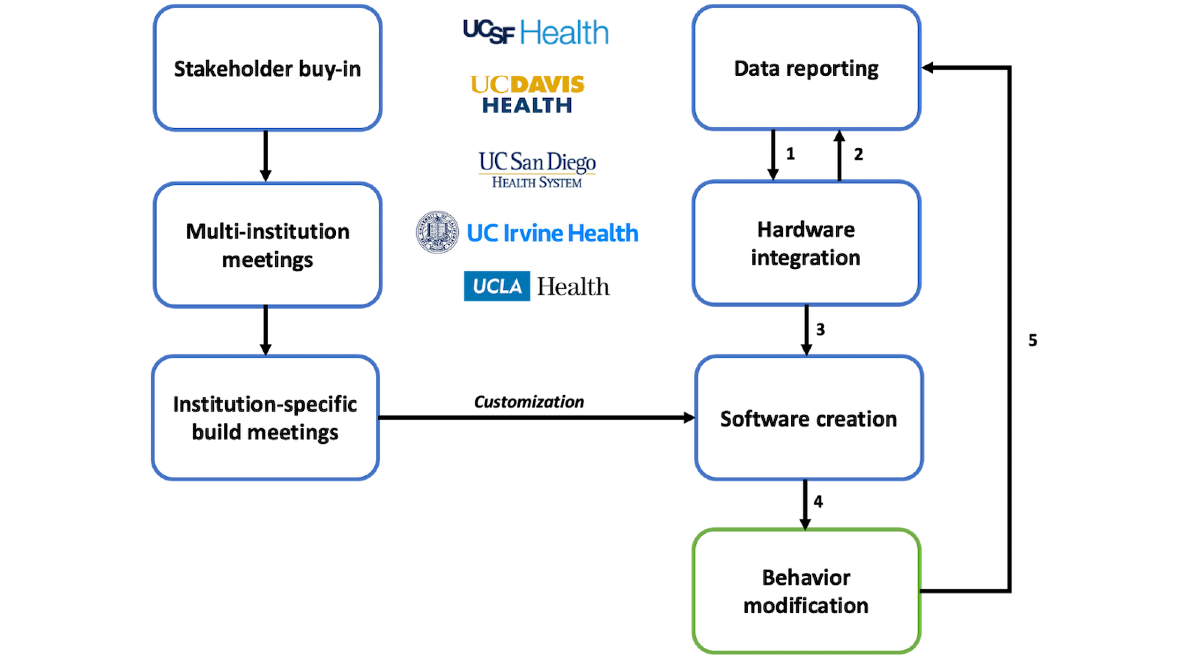
Fresh gas flow clinical decision support (CDS) toolkit implementation design. Major steps in launching a fresh gas flow CDS system at multiple institutions; step 1: based on data reporting, determine whether all necessary information is being captured in the electronic health record (EHR); step 2: if anesthesia hardware (eg, ventilators) does not transmit necessary reporting data, work with institution engineers to capture this in the EHR for data reporting and future CDS creation; step 3: CDS software design based on device data goals and institution-specific goals as framework; step 4: implementation of CDS system (with institution-specific modifications) within the EHR promotes behavior modification and subsequent reduction of anesthetic gas use; and step 5: modified clinician behavior generates additional data that can guide adjustments to the CDS system.

In early 2018 at UCSF, a committee of clinical informaticians, anesthesia professionals, and a physician sustainability champion convened to develop a simple and transferable IT solution to reduce FGF and, in turn, reduce the carbon footprint of ORs. The result was a CDS alert that worked within the Best Practice Advisory (BPA) framework of the Epic EHR (Epic Systems Corporation) to track real-time FGF and prompt anesthesia professionals to reduce FGF when the rate exceeded a defined threshold. The project was presented to departmental informatics and institution medical executive committees and adapted based on their feedback. In August 2018, the CDS alert launched within UCSF’s ORs, and subsequent volatile anesthetic waste reduction was validated [22]. In January 2021, the UC Office of the President sponsored a committee to develop and formalize the FGF CDS toolkit and launch it UC Health wide. Figure 1 outlines the implementation design of the FGF CDS toolkit.

### Ethical Considerations

As part of a prior research study analyzing the effectiveness of the FGF CDS system, the Human Research Protection Program’s Institutional Review Board approval was obtained for tracking FGF and CDS alert data at UCSF (19-28183). Subsequent implementations across the other UC Health institutions were performed under the auspices of quality improvement to reduce the environmental impact of anesthetic gases.

### Toolkit Design

The implementation process was documented and assembled into an informational toolkit to facilitate uptake of the CDS system at other health care facilities. There are 4 major components of the FGF CDS toolkit: education, hardware integration, software build, and data reporting.

### Initial Assessment and Education

**Figure 2:**
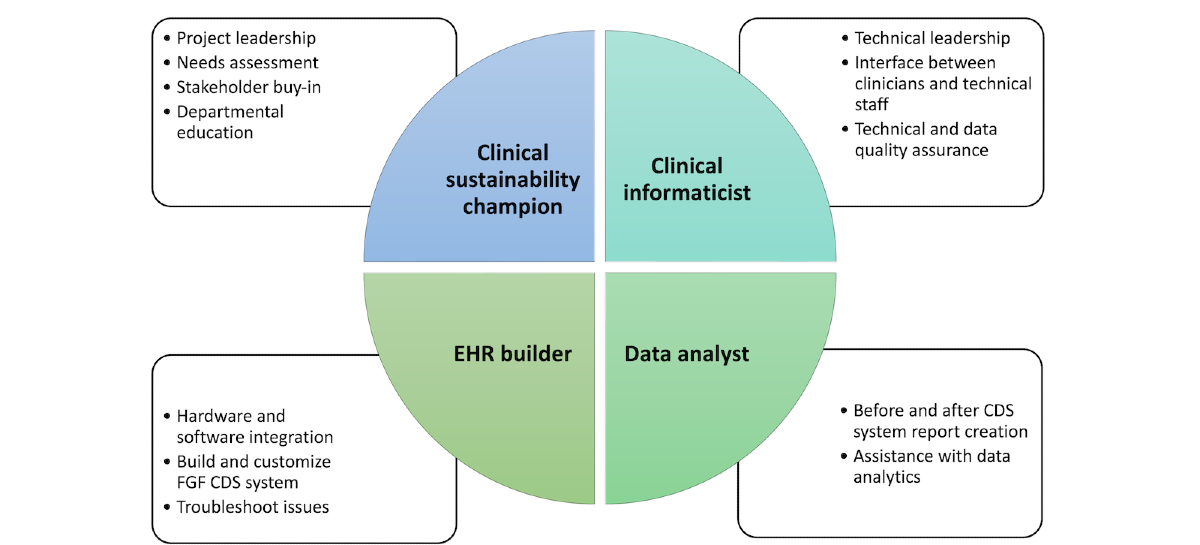
Essential roles and responsibilities for a successful fresh gas flow (FGF) clinical decision support (CDS) toolkit. EHR: electronic health record.

The initial assessment of clinician perceptions and knowledge gap regarding low FGF and subsequent targeted education to address evidence-based practice are critical before any intervention. When UCSF first launched the FGF CDS system, the sustainability and informatics leads presented at faculty meetings, trainee lectures, and grand round lectures to describe the sustainability benefits of low FGF use, the physics behind gas consumption, and details of the CDS system. This education continued in subsequent academic years after the initial FGF CDS system launch. During the first UC-wide work group meeting, the CDS system was demonstrated to informaticians, anesthesiologists, and clinical sustainability champions, along with data to support its efficacy. These site leaders, in turn, presented education materials to their trainees, faculty, and leadership at trainee lectures, faculty meetings, and departmental grand rounds. Shortly before the FGF CDS system launch at each institution, the respective clinical sustainability champions reinforced this content with additional presentations and email reminders. Essential roles and responsibilities for successful FGF CDS toolkit implementation are summarized in Figure 2.

### Hardware Integration

Before building the FGF CDS software tool, we performed an inventory of all the anesthesia ventilator machines used in ORs and their corresponding middleware outputs (eg, Capsule Medical Device Integration Platform [Capsule Technologies, Inc, a subsidiary of Philips Healthcare] used at UCSF). Multimedia Appendix 1 presents examples of the machines we use in UCSF’s ORs. Through Capsule, our AIMS was able to capture the following essential data elements for implementing the FGF CDS toolkit:

1. Set anesthetic concentration of sevoflurane, desflurane, and isoflurane (%): this value is the inspired concentration of volatile anesthetic that the practitioner sets on the anesthesia machine. This value is distinct from the actual inspired concentration delivered to a patient, which is measured by the gas analyzer.
2. FGF rate (L per minute): this value is the sum of all agents, including air, oxygen, and nitrous oxide (N_2_O).
3. End-tidal anesthetic concentration of sevoflurane, desflurane, and isoflurane (%) measured by the gas analyzer.
4. Cumulative anesthetic agent liquid consumption (mL): this volume is reported by some commercial ventilators (Multimedia Appendix 1) but may also be calculated [25].

The first 2 data elements in the aforementioned list are the *required minimum* to build a functioning FGF CDS system. The last 2 elements are useful for tracking and reporting FGF reduction impact metrics.

### Software Build

#### CDS Alert Design

At UCSF, we designed our CDS system with the goal of changing behavior without clinical disruption. First, we implemented an intraoperative CDS system with a real-time *active* (readily visible) alert to practitioners to prompt change. By contrast, a passive alert may not be readily visible (eg, one would have to scroll within the EHR to find it). Second, to prioritize patient safety and avoid disruptions to clinician workflow in a high-intensity OR setting, we chose a *noninterruptive* CDS system. The noninterruptive colored alert appears on the side of the screen, which imparts the necessary information without interrupting clinician workflow. This design is in contrast to an interruptive CDS system, which requires the clinician to respond to the alert before continuing EHR use. Our institution at UCSF felt that an interruptive CDS system would have an adverse effect on the anesthesia team, especially if an interruptive alert fired during a serious patient event. Furthermore, interruptive CDS systems may be more likely to cause alert fatigue and thus would have diminished efficacy [26,27].

#### FGF CDS Alert Rules

**Figure 3:**
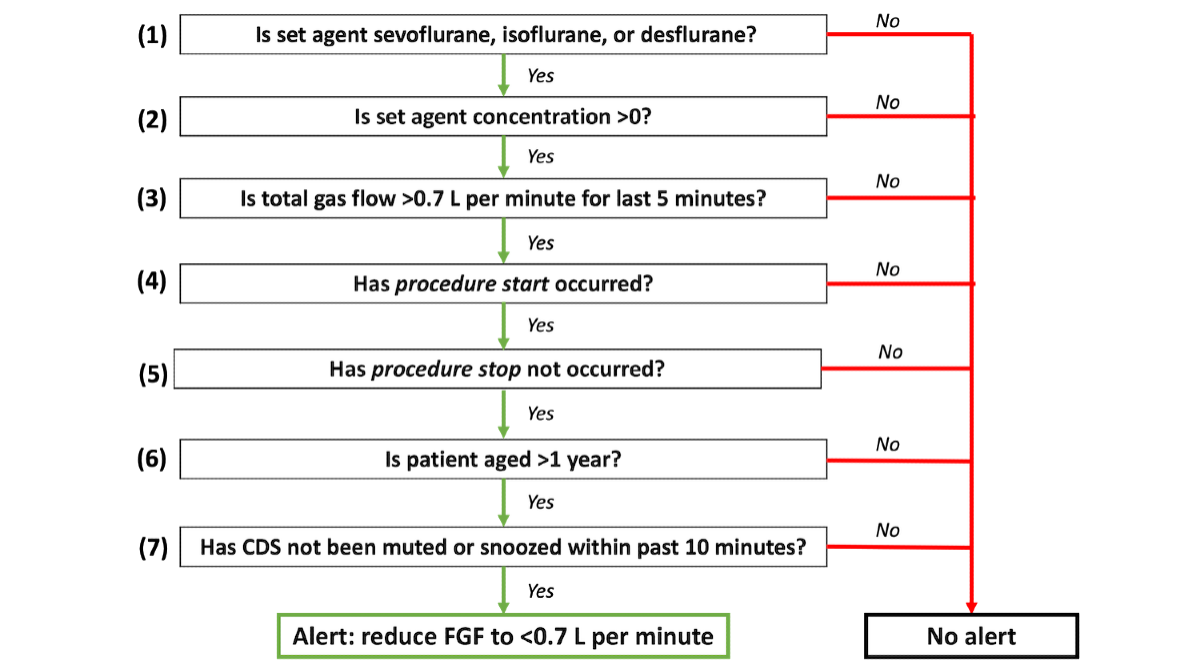
Fresh gas flow (FGF) clinical decision support (CDS) alert firing rules: these are the alert firing rules at the University of California San Francisco, based on real-time data captured in the operating room. These rules run every minute within the anesthesia information management system.

We developed a set of rules for the firing of the FGF CDS system (Textbox 2; Figure 3). These rules are evaluated every minute.

Notably, our CDS system does not activate during delivery of N_2_O without a volatile anesthetic agent but will fire if N_2_O is used in conjunction with a volatile anesthetic agent. We excluded cases with isolated use of N_2_O because it is very rare to use this agent exclusively during the maintenance phase of a procedure at our institution. However, the FGF CDS alert could easily be adapted to consider sole delivery of N_2_O.

Over multiple meetings, UCSF informaticians shared the technical specifications and report builds with the UC Office of the President’s sustainability committee leaders and EHR builders from each institution. The UC Health center-specific champions worked with their respective department leadership and IT build teams to select an FGF CDS alert type and FGF threshold to fit the needs and goals of their individual departments.

**Rules for the firing of the fresh gas flow clinical decision support system.**

- Rule 1 requires that at least one of the 3 anesthetic agents (sevoflurane, isoflurane, or desflurane) is the set agent.
- Rule 2 requires that the agent is currently in use (dial concentration is >0).
- Rule 3 requires that the set agent has a flow rate higher than the selected threshold (eg, 0.7 L per minute) for at least the last 5 consecutive minutes.
  - We chose a lookback time window of 5 minutes because we did not want abrupt, reactive changes to patient status to result in firing of the clinical decision support alert.
- Rule 4 requires that the procedure start timing event has been activated in the electronic health record.
  - This rule excludes the induction period, when the anesthesia team is typically occupied with positioning the patient, performing additional procedures, and optimizing hemodynamics. We felt that delivering an alert during this time would be disruptive to care. Furthermore, anesthesia induction frequently necessitates higher flows of anesthetic gases to quickly reach steady state plasma concentrations.
- Rule 5 requires that the procedure stop timing event has not been activated.
  - This rule helps to exclude the emergence period.
- Rule 6 requires that the patient is aged >1 year.
  - The physiology of very young patients entails unique anesthetic delivery.
- Rule 7 allows the anesthesia professional to snooze the Best Practice Advisory for a period of 10 minutes.
  - We incorporated this snooze feature and an option to turn off the clinical decision support system entirely if needed because of clinical circumstances such as circuit leak or code scenario.

### Postimplementation Reporting

**Figure 4:**
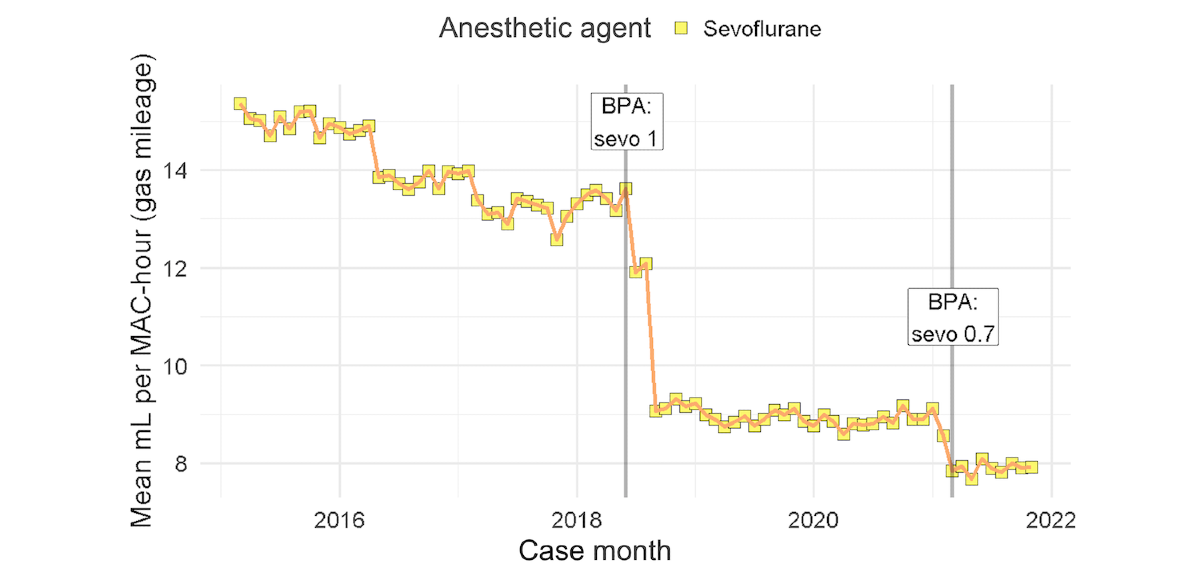
Mean mL per case of anesthetic agent per MAC-hour (the average minimum alveolar concentration [MAC] during a treatment period multiplied by the duration of treatment in hours) over time. The first mL per MAC-hour, or gas mileage, reduction occurred after the University of California San Francisco launched the fresh gas flow clinical decision support system with a rate threshold of 1 L per minute. In February 2021, the rate threshold was dropped to 0.7 L per minute and resulted in another drop in mL per MAC-hour of sevoflurane. BPA: Best Practice Advisory; sevo 0.7: sevoflurane 0.7 L per minute; sevo 1: sevoflurane 1 L per minute.

The primary outcome measure for efficiency of anesthetic administration was mL of liquid volatile anesthetic agent consumed per MAC-hour (the average MAC [minimum alveolar concentration] during a treatment period multiplied by the duration of treatment in hours) during the maintenance phase of anesthesia. This metric, mL per MAC-hour, is analogous to the inverse of miles per gallon, or *gas mileage*. To improve efficiency, one should minimize the *gas used* (volume) while maximizing the *amount of anesthesia provided* (MAC-hour). Furthermore, we can calculate total cost savings based on reduced anesthetic consumption. In addition, the age-adjusted MAC-hours of general anesthesia (as defined in Textbox 1) were calculated to (1) normalize the outcome metric related to anesthetic durations among different cases, and (2) assess the impact of our intervention on anesthetic administration practices at our institutions. Figure 4 shows the change in anesthetic gas efficiency after CDS system implementation and changes in the firing threshold at UCSF [22].

We used the following exclusion criteria for reporting:

- Locations: pediatric induction rooms, non-OR anesthesia locations (eg, interventional radiology, magnetic resonance imaging, and endoscopy) because of machine incompatibility, and labor and delivery
- Cases that used >1 volatile anesthetic agent (sevoflurane, isoflurane, or desflurane) during maintenance, defined as the presence of a single flow sheet entry for a set volatile agent concentration (%) >0 for >1 agent
- Cases with a short delivery of volatile anesthetic agent, defined as <15 minutes of recorded volatile anesthetic agent delivery per flow sheet

**Figure 5:**
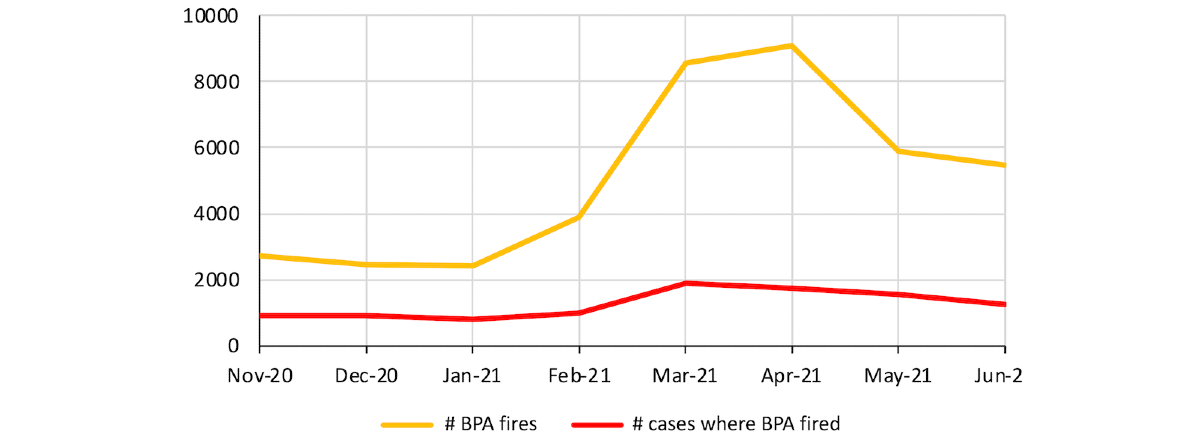
Fresh gas flow clinical decision support system firing rate after fresh gas flow threshold reduction.

With Microsoft SQL server, we extracted data directly from the Epic Clarity database. Textbox 3 shows pseudocode for how we calculated the MAC-hour per mL of volatile anesthetic agent used on a per-case or per-clinician level. The primary difference between the per-case and per-professional-per-case basis is the time windows over which the metrics are calculated. On a per-professional-per-case basis, only the portion of the maintenance phase during which the anesthesia professional was *logged in* would be taken into consideration. Thus, the maintenance phase may be split among professionals. It is also important to note that with a supervision model, multiple anesthesia professionals may have overlapping time periods because attending anesthesiologists and supervisees (certified registered nurse anesthetists and resident anesthesiologists) may hand off the case at different times. SQL code was shared with data report writers at each institution for translation into local database query language. Data on number of times the alert was fired were also extracted using Epic’s BPA Cube reporting system, providing additional insight into behavioral modification at the clinician level (Figure 5). Each institution was asked to track at least 1 month of pre–CDS system data to evaluate the impact of FGF CDS system implementation.

**Pseudocode for calculating the mL of volatile anesthetic agent per MAC-hour (the average minimum alveolar concentration [MAC] during a treatment period multiplied by the duration of treatment in hours) used on a per-case or per-clinician level.**

**Pseudocode for a given case or anesthesia professional**

- %Times based on procedure or anesthesia professional time logs
  - procedure_start=procedure or professional start time
  - procedure_stop=procedure or professional stop time
  - anesthetic_gas_stop=time of last nonzero set anesthetic delivery percentage
- %Anesthesia professional in case
  - case_duration = procedure_stop – procedure_start
- %Mark induction time period
  - induction_tp=time of first set anesthetic delivery percentage >0 to procedure_start
- %Mark maintenance time period
  - maintenance_tp=procedure_start to the earlier time of (procedure_stop or anesthetic_gas_stop)

**Pseudocode for a given inhalational anesthetic agent (isoflurane, sevoflurane, or desflurane) over case_duration**

- %Calculate volume of inhalational anesthetic liquid used during maintenance
  - set_agent_volume = cumulative anesthetic volume consumed at end of maintenance_tp – cumulative anesthetic volume consumed at end of induction_tp
- %Calculate average age-adjusted MAC during maintenance
  - age_ adjusted_MAC = average of all end-tidal anesthetic concentrations recorded during maintenace_tp / age-adjusted MAC of inhaled anesthetic agent [24]
- %Calculate MAC-hours of treatment exposure
  - MAC_hrs = age_adjusted_MAC × hours of maintenance_tp
- %Calculate mL per MAC-hour
  - ml_per_MAC_hr = set_agent_volume / MAC_hrs

## Results

On the basis of the build of the FGF CDS system at UCSF, modified versions were subsequently implemented across the UC Health network. Table 1 summarizes the individualized approaches of each UC Health system, identified as UC-A through UC-D to anonymize campuses, to implement the FGF CDS alert. The rationale for these adjustments to our noninterruptive active CDS system were based on each health system’s department-level and anesthesia professional–level feedback.

**Figure 6:**
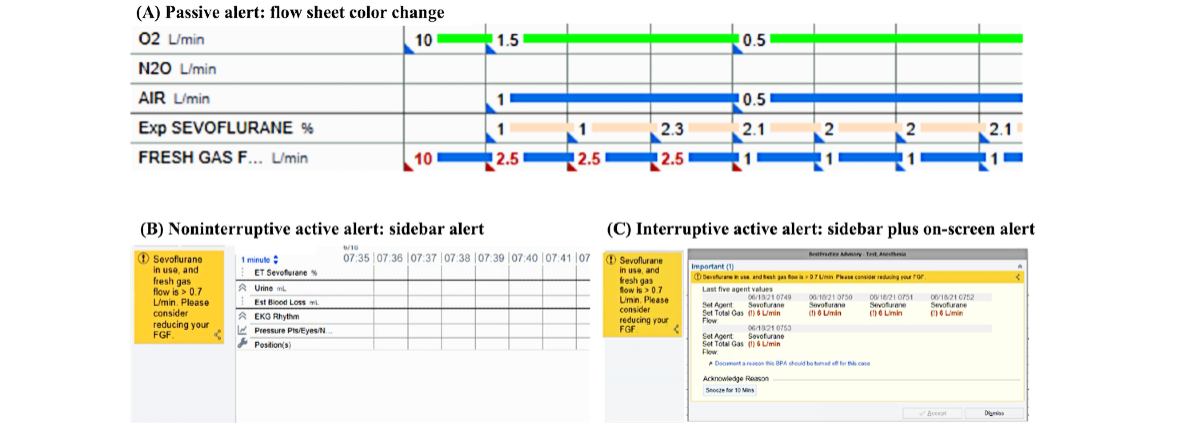
Comparison of different fresh gas flow clinical decision support alerts. (A) Depiction of a passive alert in the form of a color change in the anesthesia information management system (AIMS) flow sheet. (B) Depiction of a noninterruptive active alert in the form of a yellow sidebar alert in the AIMS interface with further details and action items on cursor hover-over. (C) Depiction of an interruptive active alert in the form of both yellow sidebar alert and on-screen pop-up window. The pop-up window must be addressed to interact with other AIMS functions.

Two health systems—UC-C and UC-D—took on the same CDS system build as UCSF: a noninterruptive active CDS with FGF alert thresholds of 0.7 L per minute and 1 L per minute, respectively. UC-A launched an interruptive active CDS system. Finally, UC-B introduced a passive CDS system with an FGF threshold of 1 L per minute. Figure 6 depicts the differences in passive versus active alert appearance: flow sheets, noninterruptive alert, and interruptive alert. With the interruptive workflow, the pop-up window (Figure 6) must be dismissed before using other EHR workflows within the AIMS.

**Table 1:** Clinical decision support (CDS) system characteristics and launch timeline.

	Health system				
	UCSF^a^	UC-A^b^	UC-B^b^	UC-C^b^	UC-D^b^
CDS system type	Active	Active	Passive	Active	Active
CDS system display	Noninterruptive	Interruptive	Flow sheet	Noninterruptive	Noninterruptive
FGF^c^ alert threshold (L per minute)	0.7	0.7	1	0.7	1
Education dates	July 2018	October 2021^d^	May 2019^e^; December 2021^d^	February 2021^e^; October 2021^d^	April 2021^e^; March 2022^d^
Launch date	September 2018	October 2021	December 2021	December 2021	May 2022

^a^UCSF: University of California San Francisco.

^b^UC-A, UC-B, UC-C, and UC-D: University of California Health system, identified as such to anonymize campuses.

^c^FGF: fresh gas flow.

^d^Best Practice Advisory tool training.

^e^Initial introduction of low-flow anesthesia.

## Discussion

### Overview

Our development and deployment of a CDS toolkit across multiple institutions demonstrates the feasibility and utility of a portable and reproducible CDS system for reducing anesthetic gas use. We describe the institutional process for implementation and how an integrated CDS system can be used to reduce the waste, cost, and carbon footprint of ORs. Our CDS toolkit can be deployed at other institutions using the popular and commercial Epic Systems EHR. Moreover, our methods can be translated into other AIMSs with identification of the proper data elements and ability to host a real-time CDS system. Widespread use of this toolkit could curb the impact of the health care system on climate change.

### Principal Findings

Our study describes the implementation of the FGF CDS system at UCSF, which was documented and assembled as an informational toolkit, and subsequent implementation at 4 other UC Health centers using the FGF CDS toolkit. Modifications to the CDS system were made after discussion from the key stakeholders at each facility. Deployment of the CDS system at all UC Health centers in this study was considered successful because the CDS system is currently in active clinical use at each center. A validation study showing that the CDS system effectively reduces anesthetic gas waste has been conducted at UCSF [22], and data collection to quantify and compare the amount of gas waste reduction among the different UC Health centers is in progress. Our study presents the FGF CDS toolkit for implementation and further shows that institutions outside of the UCSF are able to successfully modify and deploy the CDS system, making the technology accessible to the wider health care network. A primary limitation of comparable efforts to reduce anesthetic gas waste is the difficulty in transferring the technology outside of the creator institution.

Balancing benefit with burden to clinicians is always challenging when introducing any disruptive solution in health care. We were careful to create a CDS that fired with the right criteria, right information, right person, right time, and with the right intervention [28]. We also incorporated a snooze feature and a disabling feature to increase flexibility, as well as a simple and adaptable reporting structure to capture relevant data and facilitate future modifications. As demonstrated, each institution took a slightly different timeline and different approach to intervention (eg, passive vs active and noninterruptive vs interruptive).

### Barriers to Implementation

During the FGF CDS system implementation, we encountered educational, technology, and operational barriers. First, the concern for compound A formation was a reflexive response when we approached our colleagues. As this concern was anticipated from project initiation, we created educational directives to target these misconceptions before our CDS system implementation. Periodic education was required when new anesthesia staff or trainees joined the department. In addition, training and re-education was needed to establish comfort when adjunctive changes coincided with our initiative to reduce anesthetic gas waste with low FGF rates (ie, introduction of new anesthesia machines); for example, under low FGF rates, some machines may require the gas dial to be set higher than intended to overpressurize the circuit to achieve the desired MAC for general anesthesia.

From a technological perspective, some institutions had different middleware being used at different locations (eg, Capsule vs DeviceConX). The difference in middleware necessitated separate evaluations to map and discriminate the data values coming into the AIMS flow sheets and data reports. Much work and effort went into troubleshooting and fixing any errors to ensure the robustness of data.

We encountered some logistical barriers during the CDS system implementation. Our objective was to oversee a simultaneous rollout of the CDS system implementation across 4 UC Health hospitals after the implementation at UCSF. However, this goal was quite difficult given the need to accommodate the project within different work queues and IT-related priorities of each academic medical center, and the rollout was staggered among the sites. Second, some of the UC Health hospital systems only recently arrived at the final data report writing because of resource delays (eg, analyst availability) and the need for generating iterative data reports to allow for data scrutinization and to ensure data validity. Finally, shorter cases with a small maintenance phase constitute the majority of cases at some UC Health hospitals, but our CDS solution is most robust during longer cases with longer maintenance phases. A different approach or research study will be required to address the conservation of fresh gas and inhaled anesthetic at the time of induction and emergence.

### Comparison With Prior Work

To our knowledge, this is the first such FGF CDS system that has been launched across a large health network and that can be widely adopted by other institutions. Nair et al [20] demonstrated a reduction in anesthetic gas waste after implementation of a CDS system built into their proprietary EHR; however, their solution lacked ease of portability. Luria et al [29] demonstrated results similar to those of Nair et al [20] in a simulation both with and without the Low Flow Wizard (Apollo anesthesia machine; Drägerwerk AG & Co). Other studies have found evidence for the benefit of physical point-of-care reminders and educational initiatives [16,30]. Our CDS toolkit, which comprises technology combined with education and an established framework for implementation, provides an accessible route and step-by-step guide for other institutions to reduce their anesthetic gas waste.

### Limitations

There are several limitations to our FGF CDS toolkit. First, although the 5 sites where the CDS system was deployed were distinct and nonoverlapping health care systems, they were all academic centers within a single large health care network, which may limit the generalizability of our contributions; for example, institutional and anesthesia professional behavior at a small, private health care facility may be different and lead to variations in ease of implementation, available resources, and outcomes. Second, our CDS system may not be applicable in all perioperative settings. There are certain cases where high-flow inhalational agents need to be used; for example, during emergency situations, during pediatric inhalational inductions, and in select cardiothoracic surgery cases. Although our CDS system was designed to allow for such exclusion criteria and not fire the BPA under certain circumstances, it will not be effective in reducing FGF during these situations. Third, our CDS system uses the Epic EHR platform, which, although widely available, is not the platform used at all hospitals. Knowing that many institutions do have other EHR systems, we lay out the technical details and the necessary steps to import this CDS system into ORs that use other AIMSs.

### Future Directions

Studies comparing the extent of anesthetic gas waste reduction among the 5 UC Health campuses with the FGF CDS system deployed will provide additional insight into the effectiveness of various CDS system features. The CDS system and gas waste reduction will be optimized based on knowledge gained from these studies. Furthermore, we plan to support and encourage implementation of the CDS system at other health care facilities to collectively make a larger impact in anesthetic gas waste mitigation.

### Conclusions

Without compromising patient safety, health care systems should align their perioperative conservation and sustainability practices with the goals of the United Nations Intergovernmental Panel on Climate Change, whose Sixth Assessment Report unequivocally linked human influence to the rapid rates of global warming. The report further warned of dire consequences for the planet if strong, rapid, and sustained reductions in GHG emissions are not accomplished [31]. Reducing FGF has significant ecological and economic benefits in reduction of emissions from inhaled anesthetics and cost savings from less gas consumption [32].

As clinical informaticians and anesthesiologists, we can do our part to champion solutions to reduce the release of anesthetic GHG into the atmosphere. We showcased a system that achieved this aim as well as financial savings [22]. With adoption of this FGF CDS toolkit, health systems can track behavior modification, anesthetic gas use, GHG emissions, and cost per case while providing extensive opportunities for research and quality improvement. We show that EHR technologies can be used to benefit humankind by prompting hospital systems and clinicians to participate in sustainability efforts while providing high-quality care. This implementation initiative represents a crucial step in curtailing GHG emissions for the welfare of our patients and our planet alike.

As more health care professionals are becoming aware of the environmental impacts of the health care industry, we hope that the dissemination of this toolkit will facilitate the implementation of this CDS tool at other institutions for widespread adoption of low FGF nationally to advance health care decarbonization. With practices gradually evolving, anesthesia professionals should join forces through anesthesiology organizations, from regional to national societies, to advocate for *off-label* use of low FGF with sevoflurane as an evidence-based practice to counter the outdated Food and Drug Administration guidelines for anesthesia professionals [13].

## Appendix

Multimedia Appendix 1 University of California San Francisco anesthesia ventilator devices and data available via Capsule Medical Device Integration Platform (Capsule Technologies, Inc, a subsidiary of Philips Healthcare).

## Abbreviations

AIMS: anesthesia information management system
BPA: Best Practice Advisory
CDS: clinical decision support
CO2: carbon dioxide
EHR: electronic health record
FGF: fresh gas flow
GHG: greenhouse gas
MAC: minimum alveolar concentration
N2O: nitrous oxide
OR: operating room
UC: University of California
UCSF: University of California San Francisco

## Data Availability

The data underlying this paper cannot be shared publicly to protect the privacy of institutions and patients. The derived data may be shared on reasonable request to the corresponding author.
