# Supplementary material for: An Accessible Clinical Decision Support System to Curtail Anesthetic Greenhouse Gases in a Large Health Network: Implementation Study
Source: JMIR Perioper Med. 2022 Dec 8;5(1):e40831. doi: 10.2196/40831 (PMC9782391; doi:10.2196/40831)
Supplement: Multimedia Appendix 1 [file periop_v5i1e40831_app1.doc]

# SUPPLEMENTARY MATERIAL

**Supplemental Table 1: UCSF Anesthesia Ventilator Devices and Data Available via Capsule Technology Middleware**

|  | **FGF Rate** | **Vaporizer Concentration Setting (set vol %)** | **End-Tidal Gas Concentration** | **Volume (mL) of Volatile Agent** |
| --- | --- | --- | --- | --- |
| **GE**^a^ **Aisys** | **✓** | **✓** | **✓** | **✓** |
| **GE Aisys CS2** | **✓** | **✓** | **✓** | **✓** |
| **GE Avance CS2** | **✓** | **✓** | **✓** | **✓** |
| **GE Aestiva 5** | **X** | **X** | **✓** | **X** |
| **GE Aestiva 5/MRI**^b^ | **X** | **X** | **✓** | **X** |
| **Draeger Perseus A500** | **✓** | **c** | **✓** | **X**^d^ |

^a^General Electric

^b^Magnetic Resonance Imaging. These machines are compatible with MRI suites with magnets up to 3 Tesla.

^c^Depends on vaporizer

^d^ Machine displays “efficiency” of FGF
